# The association of anxiety and stress-related disorders with C-reactive protein (CRP) within UK Biobank

**DOI:** 10.1016/j.bbih.2021.100410

**Published:** 2021-12-27

**Authors:** Emma Kennedy, Claire L. Niedzwiedz

**Affiliations:** a College of Medical, Veterinary and Life Sciences, University of Glasgow, 1 Lilybank Gardens, G12 8RZ, Glasgow, Scotland, UK; b Institute of Health and Wellbeing, University of Glasgow, 1 Lilybank Gardens, G12 8RZ, Glasgow, Scotland, UK

**Keywords:** Inflammation, Anxiety, Stress, Panic, UK Biobank, C-reactive protein

## Abstract

Anxiety and stress-related disorders are both common and disabling psychiatric conditions. There are a number of hypotheses suggesting the underlying pathophysiology of these disorders, however, the exact mechanism is unknown. Inflammation has previously been linked with depression and has more recently been suggested as a possible link to anxiety aetiology. The objectives of this study are to assess the relationship between different anxiety/stress-related disorders and inflammation (measured by C-reactive protein) using the UK Biobank, and also determine whether any relationship between anxiety/stress disorders and inflammation is explained by depressive symptoms and other social and health-related factors. We utilised the UK Biobank for the sample of this study. Our sample included 353,136 participants of which 12,759 (3.61%) had a history of an anxiety (phobic, obsessive-compulsive, or other anxiety disorder including generalised anxiety and panic disorders) or stress-related disorder (including acute stress reaction, post-traumatic stress disorder and adjustment disorders). Four logistic regression models were calculated in which we tested the association between anxiety/stress disorders and C-reactive protein (CRP) >3 ​mg/L, adjusting for covariates (including age, sex, ethnicity, education level, socioeconomic deprivation, depressive symptoms, body mass index (BMI) and multimorbidity). An association was observed between other anxiety disorders (including panic and generalised anxiety disorders) and CRP (OR: 1.164 [95% CI: 1.096–1.236]). This was attenuated in models after the addition of BMI, multimorbidity and depressive symptoms. Stress/adjustment disorders followed a similar pattern of results (OR: 1.107 [95% CI: 1.040, 1.178]), with the association attenuated with the addition of BMI and multimorbidity). Phobic anxiety disorders (OR: 1.059 [95% CI: 0.896, 1.251]) and obsessive-compulsive disorders (OR: 1.299 [95% CI: 0.973, 1.733]) both showed no statistically significant results in any of the models. Our results support the hypothesis that some anxiety and stress-related disorders may be associated with high levels of inflammatory markers, as measured by CRP. Further studies are required to untangle the potential causal relationships involved.

## Highlights

• We explored the association of anxiety and stress-related disorders with C-reactive protein (CRP) using UK Biobank.
• Several disorders (e.g. generalised anxiety, panic and stress/adjustment) were associated with higher levels of CRP.
• Associations were attenuated with adjustment for health-related factors, such as multimorbidity and depressive symptoms.

## 1 Introduction

Anxiety and stress-related disorders are some of the most common and disabling types of psychiatric conditions (Kessler et al., 2005; Vos et al., 2017). They are estimated to have a 12-month prevalence of 7.3% globally as well as a pooled-lifetime prevalence of around 12.9% (Baxter et al., 2013; Steel et al., 2014). These disorders often follow a recurrent intermittent pattern throughout a person's life and can result in an overall reduced quality of life (Baxter et al., 2014; Olatunji et al., 2007). Anxiety and stress-related disorders are also associated with worsened physical health including increased risk of cardiovascular disease (Batelaan et al., 2016; Emdin et al., 2016; Song et al., 2019b), which may be independent of depression (Batelaan et al., 2016). This emphasises the need for further understanding of the biological mechanisms underlying anxiety and stress-related disorders, which may lead to improved treatments.

There are various types of anxiety and stress-related conditions, including generalised anxiety disorder (GAD), phobic anxiety disorders, obsessive-compulsive disorders (OCD) and post-traumatic stress disorder (PTSD) (World Health Organization, 2019). These present with a range of different symptoms from persistent worry and overthinking, to flashbacks observed in PTSD. Numerous studies demonstrate a familial trend in anxiety and stress-related disorders. These trends may be due to genetic factors or the family environment (Hettema et al., 2001; Telman et al., 2018). PTSD, as well as other anxiety disorders, have been associated with adverse childhood events, such as physical and sexual abuse (Afifi et al., 2012; Sareen et al., 2013). Although many risk factors have been identified to predispose someone to an anxiety/stress disorder, the biological mechanisms underpinning the disorders are not yet fully understood.

Inflammatory dysregulation has been suggested to play a role in the pathophysiology of chronic mental illness (Goldsmith et al., 2016); most considerably researched for depression (Howren et al., 2009; Mac Giollabhui et al., 2021). Several meta-analyses demonstrate that depression is associated with increased levels of peripheral inflammatory markers, such as CRP (an acute phase reactant produced by the liver in response to innate immune cytokines, notably interleukin (IL)-6 and tumor necrosis factor (TNF) (Felger et al., 2020)) and cytokines within blood (important regulators of acute and chronic inflammation) (Goldsmith et al., 2016; Howren et al., 2009; Mac Giollabhui et al., 2021). There are various reasons why the relationship between anxiety/stress-related disorders and inflammation may be important and worthy of investigation to potentially help the development of new therapeutic treatments. For example, experimentally induced stress is related to a spike in inflammatory markers, including CRP (Hamer et al., 2006). More recent neuroimaging studies have demonstrated that inflammation is found to affect anxiety-related brain regions including the amygdala, insula and anterior cingulate cortex, which may result from cytokine effects on monoamines and glutamate (Felger, 2018). Altered neurocircuitry has also been observed in people with major depressive disorder with increased peripheral inflammatory markers (Felger, 2018). Anxiety disorders are often co-morbid with depression and individuals with depression often have high levels of anxiety symptoms (Choi et al., 2020; Gaspersz et al., 2018). Therefore, there is a possibility that the two share similar neurobiological pathways. Lastly, people with anxiety and stress-related disorders have an increased risk of physical health conditions, such as metabolic disorders, cardiovascular disease and infections (Allgulander, 2016; Song et al., 2019a, 2019b; Tully et al., 2016). These are often conditions which are associated with systemic inflammation (Allgulander, 2016).

Less research relating to inflammatory markers has focused on anxiety and stress-related disorders and often only concentrates on one disorder, rather than comparing across disorders. It may be the case that specific anxiety and stress-related disorders have differing associations with inflammation, perhaps due to their individual distinct set of symptoms, but this remains unknown. A meta-analysis examining the relationship between inflammatory markers and GAD found CRP to be elevated among those with GAD compared to controls, but with a small effect size (Costello et al., 2019). Similarly, other meta-analyses of cytokine levels among people with panic disorder noted increased levels of some inflammatory markers, such as IL-6, IL-1β and IL-5, among people with panic disorder compared to control participants, but conflicting results were found for other biomarkers including IL-2, IL-12, and interferon(INF)-γ (Quagliato and Nardi, 2018). For OCD, results are also mixed with most studies finding negative results (Cosco et al., 2019). Few studies have researched stress-related disorders and those that have mainly concentrated on PTSD (Passos et al., 2015; Speer et al., 2018; Yang and Jiang, 2020). The most recent meta-analysis examining links between PTSD and immune biomarkers found levels of IL-1β, IL-2, IL-6, INF-γ, TNF-α, CRP and white blood cells were higher in PTSD than healthy controls (Yang and Jiang, 2020).

However, a common theme within previous meta-analyses is that the included studies are very heterogeneous and often have very small samples of participants (Yang and Jiang, 2020). Some included studies contain less than 10 participants and are limited to specific population groups, such as mothers of childhood cancer survivors (Glover et al., 2005) and former refugee children (Muhtz et al., 2011). Studies also often do not take into account important potential confounding variables, such as BMI and comorbidities (Costello et al., 2019). Existing research has also often not included measures of CRP (Cosco et al., 2019; Quagliato and Nardi, 2018), which is frequently collected within population-based studies and used within clinical practice as a biomarker of systemic inflammation (Felger et al., 2020). It has been demonstrated that CRP, as well as being a marker of peripheral inflammation, is also highly correlated with CRP present in cerebral spinal fluid (CSF), which is in turn associated with CSF cytokine receptors/antagonists (Felger et al., 2020).

In this study we therefore sought to address some of the above limitations of previous research. Our aim is to explore the relationship between different anxiety and stress-related disorders and inflammatory markers (measured by CRP) using UK Biobank (a large and well-characterised population-based cohort) and determine whether any relationships are explained by social and health-related factors.

## 2 Materials and methods

### 2.1 Data and study design

The UK Biobank is a large prospective cohort with baseline data from over 500,000 participants collected from 22 different assessment centres across England, Scotland and Wales during 2006–2010 (Sudlow et al., 2015). Most participants were aged 40–70 years at baseline. Data were collected on a range of factors including social and demographic factors as well as physical and mental health. The UK Biobank received ethical approval from the National Health Service National Research Ethics Service North West (16/NW/0274). We used the data in a cross-sectional study design.

### 2.2 Outcome

The primary outcome variable was inflammation as measured by high-sensitivity C-reactive protein (CRP) collected via blood samples during the baseline data collection (Fry et al., 2019; Milton et al., 2021). Circulating CRP levels were measured using high sensitivity assays of all participants at baseline. CRP was measured with the immune-turbidimetric system from the analytical platform; Beckman Coulter AU5800 (Fry et al., 2019; Milton et al., 2021). CRP was converted into a binary variable comparing those with a CRP level of ≤3 ​mg/L to those above 3 ​mg/L to indicate ‘high inflammation’, which is indicative of high adverse health risk (Felger et al., 2020; Shah et al., 2009). CRP as a continuous variable was also log-transformed due to its skewed distribution and used for sensitivity analysis. Our analysis was limited to CRP due to the lack of other available measures of inflammation within UK Biobank.

### 2.3 Exposure groups

The exposure groups included participants with a history of an anxiety or stress-related disorder and no other co-morbid mental disorder (apart from the possibility of co-morbid depression). These were documented by the codes ‘F40-3’ under the International Classification of Disease Tenth Revision (ICD-10) criteria (World Health Organization, 2019). Phobic disorders (F40) included agoraphobia, social phobias, specific phobias, and other phobic anxiety disorders. Other anxiety disorders (F41) included panic disorder, generalised anxiety disorder (GAD) and any other mixed anxiety disorder. Obsessive-compulsive disorder (F42) included all subtypes of obsessive-compulsive disorders. Stress/adjustment disorders (F43) included acute stress reaction, post-traumatic stress disorder (PTSD), adjustment disorders and other reactions to severe stress. The anxiety and stress-related disorders were identified in UK Biobank via the ‘first occurrence’ field (Category 1712). This reports the first occurrence of illness mapped to 3-character ICD-10 codes using data linked from: Read code information in the primary care data; ICD-9 and ICD-10 codes in the hospital inpatient data; ICD-10 codes in death register records; and self-reported medical condition codes reported at the baseline assessment centre visit (UK Biobank, 2019). If a participant had more than one anxiety or stress-related disorder, the earliest diagnosis code was extracted. The comparison control group included anyone without a mental or behavioural disorder (F00–F99).

### 2.4 Covariates

There were various potential factors identified in the UK Biobank database which could be accounted for. Age (years), sex (male/female) and ethnicity (white/non-white) were all recorded about each participant at baseline and were considered as potential confounding variables. During the baseline assessments, information on educational qualifications, socioeconomic deprivation, BMI and number of self-reported chronic illnesses (multimorbidity) was recorded. BMI was constructed from height and weight measured during the baseline assessment centre and categorised as underweight (<18.5); normal weight (18.5–24.9); overweight (25.0–29.9); obese (≥30.0). The number of chronic illnesses (multimorbidity) included any self-reported illnesses from a list of 43 long-term conditions (Jani et al., 2019) and we excluded mental health conditions. Education was measured as a binary variable for those with a degree and without. Deprivation was calculated using the Townsend deprivation score based on participants’ home postcode, where a higher score represented a higher level of area-based socioeconomic deprivation which was converted into quartiles for our data (Townsend, 1987). Experience of depressive symptoms was also measured at baseline via an adapted Patient Health Questionnaire (PHQ-4) (Kroenke et al., 2009; Lyall et al., 2018) and included as a continuous variable. These covariates could be considered as potentially mediating or confounding variables.

### 2.5 Statistical analysis

STATA version 16.1 (StataCorp, College Station, Texas, USA) was utilised for the statistical analysis. Firstly, we excluded those with missing data to create our final sample. Descriptive characteristics were calculated for the total sample and then characteristics were compared between those with and without an anxiety/stress-related disorder. We did a further descriptive analysis comparing participants with CRP >3 ​mg/l and ≤3 ​mg/l.

We ran four logistic regression models in order to determine the association between various anxiety/stress-related disorders and high level of CRP (>3 ​mg/L). The results from the models were displayed as odds ratios with 95% confidence intervals. Model 1 was adjusted for age and sex only. Model 2 was adjusted for age, sex, ethnicity, education and deprivation. Model 3 was adjusted for all covariates in model 2 with the addition of BMI and multimorbidity. Model 4 was adjusted for all of the variables in model 3 with the addition of the PHQ-4 scale.

For sensitivity analysis, we conducted linear regression on the same four models using log-transformed values of CRP. This analysis was used to investigate whether the pattern of results (e.g. direction of associations) observed for the logistic models were similar. A further sensitivity analysis was carried out for depressive symptoms by removing BMI and multimorbidity from model 4. This was to confirm that model 4's results were not explained solely by model 3's variables – BMI and multimorbidity. We also tested to see if either BMI or multimorbidity were impacting model 3 more by removing BMI and keeping multimorbidity and vice versa.

## 3 Results

### 3.1 Descriptive statistics

**Fig. 1:**
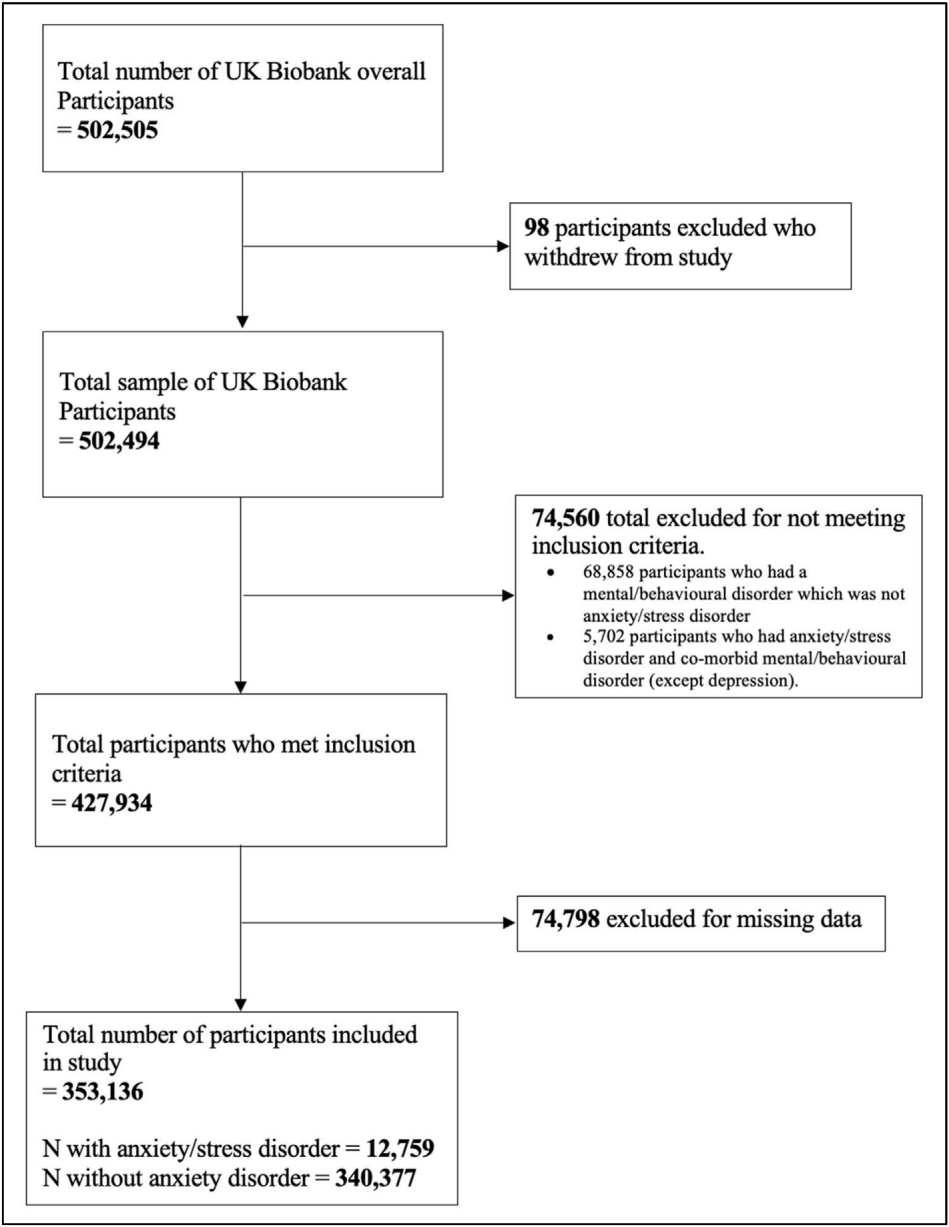
Flowchart of study participants.

The final sample used for the statistical analysis consisted of 353,136 participants of which 12,759 (3.61%) had a history of anxiety or stress-related disorders (see Fig. 1)*.* Table 1 displays the descriptive characteristics of the total sample and compares this to the anxiety/stress and control group. Comparing the anxiety/stress and control groups, the mean age was approximately the same with 56.2 in anxiety/stress and 56.6 in control groups. The levels of deprivation between the two groups were both evenly distributed and there was a slightly higher percentage of people from the anxiety/stress group in the obese category for BMI, however, this was only a 4% difference. The anxiety/stress group had a higher percentage of people with 1 or more chronic illnesses. The anxiety/stress group also had a significantly higher average PHQ-4; therefore, they presented with a higher number of depressive symptoms on average compared to the control group. Furthermore, there was a higher average CRP level in the anxiety/stress group. There were slightly more participants with an anxiety/stress disorder with a CRP level >3 ​mg/L group (4.03%) compared with 3.5% among participants with CRP ≤3 ​mg/L (Table 2).

**Table 1:** Characteristics of the sample.

Characteristics	Overall Sample (n ​= ​353,136) Number (%)	Anxiety/stress Disorder Group [ICD-10: F40-3] (n ​= ​12,759) Number (%)	Control Group (n ​= ​340,377) Number (%)
Age group (years)			
40-44	35,992 (10.19)	1,260 (9.88)	34,732 (10.20)
45-49	45,777 (12.96)	1,670 (13.09)	44,107 (12.96)
50-54	53,094 (15.04)	2,113 (16.56)	50,981 (14.98)
55-59	63,898 (18.09)	2,530 (19.83)	61,368 (18.03)
60-64	86,602 (24.52)	3,198 (25.06)	83,404 (24.50)
65-69	66,065 (18.71)	1,942 (15.22)	64,123 (18.84)
70>	1,708 (0.48)	46 (0.36)	1,662 (0.49)
Mean age (SD)	56.60 (8.08)	56.21 (7.78)	56.61 (8.09)
Sex			
Female	188,036 (53.25)	8,324 (65.24)	179,712 (52.80)
Male	165,100 (46.75)	4,435 (34.76)	160,665 (47.20)
Ethnicity			
White	337,222 (95.49)	aWhite: 12,322 (96.57)	aWhite: 324,900 (95.45)
Mixed	1,892 (0.54)	Non-white: 437 (3.43)	Non-white: 15,477 (4.55)
South Asian	5,468 (1.55)		
Black	4,878 (1.38)		
Chinese	1,037 (0.29)		
Other	2,639 (0.75)		
Education			
Degree	123,184 (34.88)	4,065 (31.86)	119,119 (35.00)
Non-degree	229,952 (65.12)	8,694 (68.14)	221,258 (65.00)
Deprivation			
Q1 (most advantaged)	94,337 (26.71)	3,262 (25.57)	91,075 (26.76)
Q2	91,894 (26.02)	3,235 (25.35)	88,659 (26.05)
Q3	88,596 (25.09)	3,343 (26.20)	85,235 (25.05)
Q4	78,309 (22.18)	2,919 (22.88)	75,390 (22.15)
BMI			
Underweight (<18.5)	1,648 (0.47)	59 (0.46)	1,589 (0.47)
Normal weight (18.5–24.9)	118,348 (33.51)	3,979 (31.19)	114,369 (33.60)
Overweight (25.0–29.9)	151,982 (43.04)	5,299 (41.53)	146,683 (43.09)
Obese (≥30.0)	81,158 (22.98)	3,422 (26.82)	77,736 (22.84)
No. of Chronic illness			
0	134,479 (38.08)	3,844 (30.13)	130,635 (38.38)
1	118,698 (33.61)	4,195 (32.88)	114,503 (33.64)
2	62,582 (17.72)	2,624 (20.57)	59,958 (17.62)
3	25,263 (7.15)	1,302 (10.20)	23,961 (7.04)
4	8,397 (2.38)	504 (3.95)	7,893 (2.32)
5+	3,717 (1.05)	290 (2.27)	3,427 (1.01)
PHQ-4 (scale)			
Mean (SD)	1.38 (1.84)	2.49 (2.67)	1.34 (1.79)
CRP level >3 ​mg/L			
Yes	74,575 (21.12)	3,008 (23.58)	71,567 (21.03)
No	278,561 (78.88)	9,751 (76.42)	268,810 (78.97)
CRP level Mean (mg/L)			
Normal Mean (CI)	2.47 (2.46–2.49)	2.67 (2.59–2.74)	2.47 (2.45–2.48)
Geometric Mean (CI)	1.33 (1.33–1.34)	1.44 (1.41–1.46)	1.33 (1.32–1.33)
Anxiety/stress-related disorder			
None	340,377 (96.39)		
Phobic anxiety disorder (F40)	784 (0.22)		
Other anxiety disorder (F41)	5,972 (1.69)		
Obsessive-compulsive disorder (F42)	249 (0.07)		
Stress/adjustment disorder (F43)	5,754 (1.63)		

Key: CI ​= ​95% confidence intervals. SD ​= ​standard deviations.

a Ethnicity has been dichotomised to white/non-white due to small numbers.

**Table 2:** Characteristics of participants with low-moderate (≤3 ​mg/L) and high levels of CRP (>3 ​mg/L).

Characteristic	Low-moderate CRP (%) CRP ≤3 (n ​= ​278,561)	High CRP (%) CRP >3 (n ​= ​74,575)
Age group (years)		
40-44	29,939 (10.75)	6,053 (8.12)
45-49	37,790 (13.57)	7,987 (10.71)
50-54	42,583 (15.29)	10,511 (14.09)
55-59	50,588 (18.16)	13,310 (17.85)
60-64	66,733 (23.96)	19,869 (26.64)
65-69	49,649 (17.82	16,416 (22.01)
70>	1,279 (0.46)	429 (0.58)
Mean age (SD)	56.32 (8.11)	57.64 (7.87)
Sex		
Female	144,626 (51.92)	43,410 (58.21)
Male	133,835 (48.08)	31,165 (41.79)
Ethnicity		
White	266,311 (95.60)	70,911 (95.09)
Non-White	12,250 (4.40)	3,664 (4.91)
Education		
Degree	102,786 (36.90)	20,398 (27.35)
Non-degree	175,775 (63.10)	54,177 (72.65)
Deprivation		
Q1 (most advantaged)	76,696 (27.53)	17,641 (23.66)
Q2	73,465 (26.37)	18,429 (24.71)
Q3	69,775 (25.05)	18,821 (25.24)
Q4	58,625 (21.05)	19,684 (26.39)
BMI		
Underweight (<18.5)	1,510 (0.54)	138 (0.19)
Normal weight (18.5–24.9)	106,224 (38.13)	12,125 (16.26)
Overweight (25.0–29.9)	123,097 (44.19)	28,885 (38.73)
Obese (≥30.0)	47,731 (17.13)	33,427 (44.82)
No. of Chronic illness		
0	113,549 (40.76)	20,830 (28.07)
1	93,954 (33.73)	24,744 (33.18)
2	46,319 (16.63)	16,263 (21.81)
3	17,335 (6.22)	7,928 (10.63)
4	5,279 (1.90)	3,118 (4.18)
5+	2,125 (0.76)	1,592 (2.13)
PHQ-4 (scale)		
Mean (SD)	1.32 (1.79)	1.59 (2.01)
Anxiety/stress-related disorder		
None	268,810 (96.50)	71.567 (95.97)
Phobic anxiety disorder (F40)	603 (0.22)	181 (0.24)
Other anxiety disorder (F41)	4,529 (1.63)	1,443 (1.93)
Obsessive-compulsive disorder (F42)	187 (0.07)	62 (0.08)
Stress/adjustment disorder (F43)	4,432 (1.59)	1,322 (1.77)
Anxiety/stress-related disorder		
Yes	9,751 (3.50)	3,008 (4.03)
No	268,810 (96.50)	71,567 (95.97)

SD = Standard deviation.

### 3.2 Logistic regression results

**Fig. 2:**
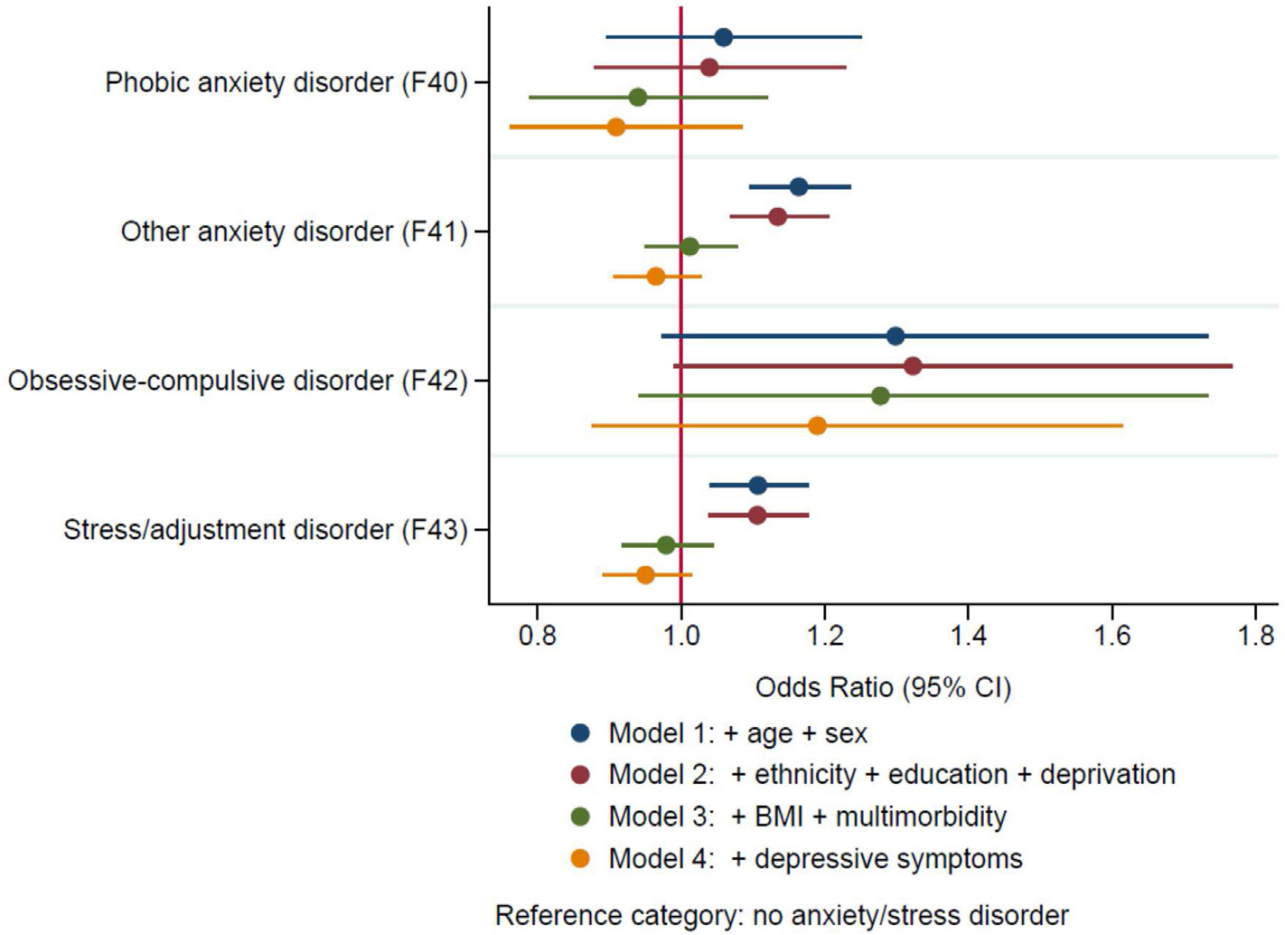
The association between anxiety/stress-related disorders and C-reactive protein (CRP).

The “other anxiety disorders” group were 1.164 times more likely to have a CRP above 3 ​mg/L in model 1 with a p value ​< ​0.001 (Table 3). This was slightly attenuated in model 2 which was adjusted for social factors (ethnicity, education and deprivation), with an OR 1.134 [95% CI: 1.068–1.205]. These were further attenuated (and no statistically significant results were seen) in models 3 (adjusted for health-related factors e.g. BMI and multimorbidity) and 4 (adjusted for current depressive symptoms) (Fig. 2). Stress/adjustment disorders demonstrated a similar pattern with increased odds in models 1 and 2, however, this was attenuated in models 3 and 4. Model 1 showed a 1.107 increased likelihood of >3 ​mg/L CRP in stress/adjustment disorders with p value ​< ​0.01 and model 2 showed an OR of 1.106 [95% CI: 1.039–1.177]. Obsessive-compulsive disorder showed the strongest positive OR in each of the 4 models. Although, both obsessive-compulsive disorders and phobic anxiety disorders showed no statistically significant results in any of the models (Fig. 2).

**Table 3:** Logistic models for the association between anxiety/stress-related disorders and high CRP (>3 ​mg/L).

Characteristics	CRP level above 3 ​mg/L Odds Ratio [95% confidence intervals]			
	Model 1	Model 2	Model 3	Model 4
Anxiety/stress-related disorder				
None (reference) (n ​= ​268,810)				
Phobic anxiety disorder (F40) (n ​= ​603)	1.059 [0.896, 1.251]	1.039 [0.879, 1.229]	0.940 [0.788, 1.121]	0.909 [0.762, 1.085]
Other anxiety disorder (F41) (n ​= ​4,529)	1.164∗∗∗ [1.096, 1.236]	1.134∗∗∗ [1.068, 1.205]	1.012 [0.95, 1.078]	0.965 [0.905, 1.029]
Obsessive-compulsive disorder (F42) (n ​= ​187)	1.299 [0.973, 1.733]	1.323 [0.990, 1.768]	1.277 [0.941, 1.733]	1.190 [0.876, 1.615]
Stress/adjustment disorder (F43) (n ​= ​4,432)	1.107∗∗ [1.040, 1.178]	1.106∗∗ [1.039, 1.177]	0.979 [0.917, 1.045]	0.950 [0.890, 1.015]
Age group (years)				
40–44 (reference)				
45-49	1.040∗ [1.002, 1.078]	1.041∗ [1.003, 1.080]	0.986 [0.949, 1.025]	0.989 [0.952, 1.028]
50-54	1.211∗∗∗ [1.17, 1.254]	1.222∗∗∗ [1.18, 1.266]	1.073 ∗∗∗ [1.034, 1.113]	1.082∗∗∗ [1.043, 1.122]
55-59	1.294∗∗∗ [1.251, 1.339]	1.310∗∗∗ [1.266, 1.355]	1.104∗∗∗ [1.065, 1.144]	1.122∗∗∗ [1.083, 1.163]
60-64	1.471∗∗∗ [1.424, 1.518]	1.460∗∗∗ [1.414, 1.508]	1.191∗∗∗ [1.151, 1.232]	1.226∗∗∗ [1.184, 1.269]
65-69	1.653∗∗∗ [1.599, 1.708]	1.605∗∗∗ [1.552, 1.659]	1.277∗∗∗ [1.233, 1.323]	1.323∗∗∗ [1.276, 1.371]
70>	1.681∗∗∗ [1.502, 1.883]	1.628∗∗∗ [1.454, 1.824]	1.291∗∗∗ [1.146, 1.455]	1.342∗∗∗ [1.191, 1.512]
Sex				
Female (reference)				
Male	0.769∗∗∗ [0.756, 0.781]	0.777∗∗∗ [0.764, 0.790]	0.674∗∗∗ [0.662, 0.686]	0.679∗∗∗ [0.667, 0.691]
Ethnicity				
White (reference)				
Non-white		1.135∗∗∗ [1.092, 1.181]	1.060∗∗ [1.017, 1.104]	1.035 [0.994, 1.079]
Education				
Degree (reference)				
Non-degree		1.468∗∗∗ [1.442, 1.495]	1.242∗∗∗ [1.219, 1.266]	1.235∗∗∗ [1.212, 1.259]
Deprivation				
Q1 (reference category)				
Q2		1.076∗∗∗ [1.051, 1.101]	1.043∗∗∗ [1.018, 1.068]	1.041∗∗∗ [1.017, 1.067]
Q3		1.172∗∗∗ [1.146, 1.200]	1.097∗∗∗ [1.070, 1.123]	1.091∗∗∗ [1.065, 1.118]
Q4		1.469∗∗∗ [1.435, 1.504]	1.270∗∗∗ [1.239, 1.301]	1.252∗∗∗ [1.221, 1.283]
BMI				
Underweight (<18.5)			0.742∗∗∗ [0.622, 0.884]	0.732∗∗∗ [0.614, 0.873]
Normal weight (18.5–24.9) (reference)				
Overweight (25.0–29.9)			2.072∗∗∗ [2.024, 2.121]	2.071∗∗∗ [2.023, 2.120]
Obese (≥30.0)			5.660∗∗∗ [5.524, 5.799]	5.626∗∗∗ [5.491, 5.765]
No. of Chronic illness				
0 (reference)				
1			1.219∗∗∗ [1.193, 1.246]	1.208∗∗∗ [1.183, 1.235]
2			1.401∗∗∗ [1.366, 1.436]	1.377∗∗∗ [1.343, 1.412]
3			1.614∗∗∗ [1.562, 1.668]	1.571∗∗∗ [1.520, 1.624]
4			1.885∗∗∗ [1.793, 1.982]	1.815∗∗∗ [1.726, 1.909]
5+			2.152∗∗∗ [2.004, 2.311]	2.035∗∗∗ [1.894, 2.186]
PHQ-4 (scale)				1.037∗∗∗ [1.032, 1.042]

Model 1: adjusted for: age, sex; Model 2: Model 1 ​+ ​ethnicity, education and deprivation; Model 3: Model 2 ​+ ​BMI, multimorbidity Model 4: Model 3 ​+ ​PHQ-4.

∗p value ​< ​0.05.

∗∗p value ​< ​0.01.

∗∗∗p value ​< ​0.001.

The overweight and obese BMI groups showed an increased likelihood of CRP >3 ​mg/L in both model 3 and 4. For instance, the obese category showed an OR of 5.660 [95% CI: 5.524–5.799] in model 3. Multimorbidity also appeared to have a large impact in models 3 and 4. As the number of chronic illnesses increased the odds ratio for high CRP increased too. Higher depressive symptoms (measured by PHQ-4 scale) were also related to increased likelihood of high CRP.

### 3.3 Sensitivity analysis

Similar patterns of results were observed in the linear regression models, as seen in Table 4. Phobic anxiety disorders showed no significant association with CRP within any of the 4 models, which was similar to the results seen in the logistic regression. Obsessive-compulsive disorders were similar with none of the results showing statistical significance, however, all showed a positive association with CRP. “Other anxiety disorders” again showed statistically significant results in both models 1 and 2, but not models 3 and 4. Stress/adjustment disorders in models 1 and 2 were both associated with CRP with p values ​< ​0.001. Similar to “other anxiety disorders”, the health-related factors appeared to attenuate the associations.

**Table 4:** Linear models for the association between anxiety/stress-related disorders and CRP level.

Characteristics	CRP (continuous) Regression Coefficient [95% confidence intervals]			
	Model 1	Model 2	Model 3	Model 4
Anxiety/stress-related disorder				
None (reference category)				
Phobic anxiety disorder (F40)	0.038 [-0.035, 0.111]	0.027 [-0.045, 0.100]	−0.025 [-0.092, 0.041]	−0.040 [-0.106, 0.027]
Other anxiety disorder (F41)	0.081∗∗∗ [0.055, 0.108]	0.066∗∗∗ [0.040, 0.093]	0.005 [-0.020, 0.029]	−0.016 [-0.040, 0.009]
Obsessive-compulsive disorder (F42)	0.114 [-0.016, 0.243]	0.123 [-0.005, 0.251]	0.092 [-0.026, 0.211]	0.060 [-0.058, 0.179]
Stress/adjustment disorder (F43)	0.078 ∗∗∗ [0.051, 0.105]	0.076∗∗∗ [0.049, 0.103]	0.007 [-0.018, 0.032]	−0.006 [-0.031, 0.019]
Age group (years)				
40–44 (reference)				
45-49	0.044∗∗∗ [0.030, 0.059]	0.043∗∗∗ [0.029, 0.057]	0.014∗ [0.000, 0.027]	0.015∗ [0.002, 0.028]
50-54	0.152∗∗∗ [0.138, 0.166]	0.152∗∗∗ [0.138, 0.166]	0.079∗∗∗ [0.066, 0.092]	0.082∗∗∗ [0.070, 0.095]
55-59	0.218∗∗∗ [0.204, 0.231]	0.217∗∗∗ [0.204, 0.231]	0.121∗∗∗ [0.108, 0.133]	0.128∗∗∗ [0.115, 0.140]
60-64	0.314∗∗∗ [0.301, 0.327]	0.299∗∗∗ [0.286, 0.312]	0.179∗∗∗ [0.167, 0.191]	0.191∗∗∗ [0.179, 0.203]
65-69	0.409∗∗∗[0.396, 0.423]	0.380∗∗∗ [0.367, 0.394]	0.243∗∗∗ [0.231, 0.256]	0.258∗∗∗ [0.246, 0.271]
70>	0.473∗∗∗ [0.422, 0.524]	0.441∗∗∗ [0.391, 0.492]	0.303∗∗∗ [0.257, 0.349]	0.319∗∗∗ [0.273, 0.366]
Sex				
Female (reference)				
Male	−0.063∗∗∗ [-0.070,-0.056]	−0.055∗∗∗ [-0.062,-0.048]	−0.148∗∗∗ [-0.154,-0.142]	−0.145∗∗∗ [-0.151,-0.138]
Ethnicity				
White (reference)				
Non-white		0.059∗∗∗ [0.042, 0.075]	0.017∗ [0.002, 0.032]	0.007 [-0.009, 0.022]
Education				
Degree (reference)				
Non-degree		0.235∗∗∗ [0.228, 0.242]	0.126∗∗∗ [0.120, 0.133]	0.124∗∗∗ [0.117, 0.131]
Deprivation				
Q1 (reference)				
Q2		0.038∗∗∗ [0.028, 0.047]	0.020∗∗∗ [0.011, 0.028]	0.019∗∗∗ [0.010, 0.028]
Q3		0.067∗∗∗ [0.058, 0.077]	0.030∗∗∗ [0.021, 0.039]	0.028∗∗∗ [0.019, 0.037]
Q4		0.173∗∗∗ [0.163, 0.183]	0.092∗∗∗ [0.083, 0.101]	0.086∗∗∗ [0.077, 0.095]
BMI				
Underweight (<18.5)			−0.488∗∗∗ [-0.534,-0.441]	−0.493∗∗∗ [-0.539,-0.447]
Normal weight (18.5–24.9) (reference)				
Overweight (25.0–29.9)			0.488∗∗∗ [0.481, 0.496]	0.488∗∗∗ [0.481, 0.495]
Obese (≥30.0)			1.031∗∗∗ [1.022, 1.039]	1.027∗∗∗ [1.019, 1.036]
No. of Chronic illness				
0 (reference)				
1			0.095∗∗∗ [0.087, 0.102]	0.091∗∗∗ [0.083, 0.098]
2			0.159∗∗∗ [0.149, 0.168]	0.151∗∗∗ [0.142, 0.161]
3			0.229∗∗∗ [0.215, 0.242]	0.217∗∗∗ [0.203, 0.230]
4			0.311∗∗∗ [0.290, 0.332]	0.294∗∗∗ [0.273, 0.316]
5+			0.377∗∗∗ [0.346, 0.409]	0.352∗∗∗ [0.321, 0.384]
PHQ-4 (scale)				0.016∗∗∗ [0.015, 0.018]

CRP was log-transformed prior to analysis.

∗p value <0.05.

∗∗p value ​< ​0.01.

∗∗∗p value ​< ​0.001.

Further sensitivity analyses were conducted to determine which covariates were having the biggest impact on models 3 and 4. From the analysis in Table 5 we can see that BMI, multimorbidity and depressive symptoms all had an impact on the results. In the model without multimorbidity compared to the original model 3, “other anxiety disorders” showed a significant result. However, all of the other results were similar to the model 3, therefore, suggesting all the covariates contributed to the associations observed.

**Table 5:** Sensitivity analysis: Logistic regression exploring the influence of BMI, multimorbidity and depressive symptoms on the association between anxiety/stress-related disorders and high CRP.

Anxiety/stress-related disorder	CRP level above 3 ​mg/L Odds Ratio [95% confidence intervals]			
	Model 3	Model 3 without Multimorbidity	Model 3 without BMI	Model 4 without BMI and Multimorbidity
None (reference category)				
Phobic Anxiety Disorder	0.940 [0.788, 1.121]	0.981 [0.823, 1.169]	0.961 [0.811, 1.138]	0.968 [0.818, 1.146]
Other Anxiety Disorder	1.012 [0.95, 1.078]	1.072∗ [1.006, 1.142]	1.030 [0.969, 1.095]	1.027 [0.966, 1.091]
Obsessive-compulsive Disorder	1.277 [0.941, 1.733]	1.311 [0.968, 1.777]	1.265 [0.943, 1.695]	1.143 [0.854, 1.530]
Stress/adjustment Disorder	0.979 [0.917, 1.045]	1.011 [0.948, 1.080]	1.042 [0.978, 1.109]	1.040 [0.976, 1.107]

Model 3 adjusted for age, sex, ethnicity, education, deprivation, BMI and multimorbidity.

Model 4 adjusted for age, sex, ethnicity, education, deprivation, BMI, multimorbidity and PHQ-4.

∗p value ​< ​0.05.

## 4 Discussion

This study investigated associations of anxiety and stress-related disorders with inflammation, specifically C-reactive protein (CRP) levels. We found associations between stress/adjustment disorders (F43) and “other anxiety disorders” (F41) (e.g. generalised anxiety disorder, panic disorders) and CRP after accounting for the following potential confounders: age, sex, ethnicity, deprivation and education level. However, none of the associations remained after adjusting for health-related factors which included: BMI, multimorbidity and depressive symptoms. Obsessive-compulsive disorders and phobic anxiety disorders showed no statistically significant associations with CRP in any of the four models.

Our findings correlate with other studies in terms of the direction of associations (Costello et al., 2019). Results for GAD are similar to other studies which demonstrate the association between GAD and CRP was attenuated by BMI and other factors (Copeland et al., 2012). Those anxiety disorders that were not found to be associated (obsessive-compulsive disorder and phobic anxiety), also showed the same direction of correlation, but within these categories there were smaller sample sizes. This suggests that they may show similar findings if they had enough power to do so. Our findings agree with the positive relationship seen in stress disorders, as many studies have found an association between inflammation and PTSD (Passos et al., 2015). However, in our study this finding is not seen after adjusting for health-related factors and depressive symptoms.

Although anxiety has not been well established as a factor associated with increased inflammation, depression has been comprehensively studied (Mac Giollabhui et al., 2021). As depression is highly co-morbid with anxiety there is speculation that they might have similar underlying neurobiological mechanisms, which potentially involves the inflammatory pathway (Lamers et al., 2011). In this study, we found no statistically significant relationships after adjusting for both health-related factors and depressive symptoms. This suggests that the relationship between anxiety and inflammation is partly explained by depressive symptoms and health-related factors such as BMI and multimorbidity. Therefore, we cannot conclude that the relationship between anxiety disorders and inflammation is independent to depression and the other factors investigated. Furthermore, as the relationship between depression and inflammation has been suggested to be bidirectional, this may also be the case for anxiety/stress and inflammation (Beurel et al., 2020). For instance, anxiety could potentially increase inflammation and in turn, heightened inflammation could also be an inducer of anxiety.

### 4.1 Strengths and limitations

This study provides a large, well-characterised population-based sample which we believe may increase the accuracy of the results. A further strength is the inclusion of variables that may confound the relationships of interest. Furthermore, we explored several anxiety/stress-related disorder categories within our study which provides a comparison of each disorder's association with inflammation and adds depth to the research.

There are a number of limitations which must also be acknowledged. Firstly, within this study, we only examined one inflammatory marker (CRP), due to its good availability within large population-based cohort studies and clinical relevance. Having a wider range of inflammatory markers may give clarity to the potential underlying pathophysiology involved (Costello et al., 2019; Parsons et al., 2021; Vogelzangs et al., 2013). We adjusted for a large set of possible confounding factors, however, unmeasured lifestyle or health factors may also explain some of the associations found. We did not include health behaviours related to anxiety that could potentially be mediators of the relationship between anxiety/stress disorders and inflammation, such as smoking and alcohol consumption. Diagnoses of anxiety/stress-related disorders were obtained via medical records and self-report. Those that only self-reported anxiety may be more unreliable as a proportion of those with self-reported anxiety diagnoses had no link to GP records and this could lead to an underestimation of the observed associations. GP and hospital recorded diagnoses are more likely to reflect severe anxiety that was treated by health services. The reliance on a diagnostic approach is also a limitation; different results may be obtained by examining specific anxiety symptoms and their severity. We also categorised disorders based on broad ICD-10 codes which meant we were unable to examine very specific disorders in isolation. Furthermore, it was not possible to longitudinally investigate the association between changes in anxiety/stress with changes in CRP levels. UK Biobank data also has various limitations. There is a significant selection bias within the data, which makes it unlikely to represent the general population in terms of certain characteristics, such as ethnicity, general health and socioeconomic position as healthy, affluent and white participants were more likely to participate (Fry et al., 2017).

The largely cross-sectional nature of this study makes it difficult to explore whether inflammation is a potential cause of anxiety/stress or if they result in an inflammatory response. Due to this, we cannot make any inferences about the causal direction of the associations. More recent research has sought to address this issue using a Mendelian randomization (MR) approach (Milaneschi et al., 2021; Ye et al., 2021). Using UK Biobank data to assess the relationship between symptoms of anxiety and concentrations of CRP, as well as genetic variants associated with CRP and IL-6 receptor gene regions, it was found that increased anxiety symptoms were related to higher levels of CRP in a dose-response manner (especially among females), but the MR analysis suggested the opposite to that expected, higher CRP was protective against anxiety (Ye et al., 2021). Similar to our findings, the associations observed using circulating CRP concentrations appeared to be explained by depressive symptoms. Further exploration of specific symptoms of anxiety within the UK Biobank cohort and the Netherlands Study of Depression and Anxiety (NESDA) demonstrated higher circulating CRP to be associated with irritability and worrying control, but results were less consistent in MR analyses and stronger associations were found for symptoms of depression (Milaneschi et al., 2021).

However, studies which adopt a MR approach are often limited by the exclusion of non-European ancestry populations (Milaneschi et al., 2021; Ye et al., 2021). Replication, as well as triangulation of methods (including experimental and observational studies) are required to further elucidate the pathways involved for all population groups. By following people from a young age (before the onset of anxiety and depressive symptoms) via a longitudinal study, any potential changes in inflammation that may arise with the onset of anxiety could be monitored. This research would help determine whether immune dysregulation is a precursor or the result of anxiety – or if this is a bidirectional pathway. Bidirectional MR studies would also help elucidate this. There remains a vast amount of research required to establish the underlying pathophysiology of anxiety and stress-related disorders in order to improve the current treatment options available for the disorders.

## 5 Conclusion

In conclusion, our results support the hypothesis that some anxiety and stress-related disorders (including panic disorder, stress/adjustment disorders and GAD) may be associated with high levels of inflammation, as measured by CRP. However, the associations were attenuated by health-related factors including multimorbidity, BMI and depressive symptoms. Further large birth cohorts, Mendelian randomization and experimental studies are required to untangle the potential causal relationships and mechanisms involved.

## Contributions

EK and CLN conceived the idea for the study. EK and CLN designed the study. EK conducted the analysis, with assistance from CLN. EK drafted the manuscript and it was critically revised by CLN.

## Funding

CLN acknowledges funding from the 10.13039/501100000265Medical Research Council (MR/R024774/1) and a Lord Kelvin/Adam Smith Fellowship. The funders had no role in the study design, data collection, data analysis, data interpretation, or writing of the report.

## Data availability

Data are available from UK Biobank (https://www.ukbiobank.ac.uk/), but restrictions apply to their availability. The data were used under licence for the current study and so are not publicly available, but are available from the authors upon reasonable request and with permission of UK Biobank.

## Declaration of competing interest

None.
